# Fertility desires, family planning use and pregnancy experience: longitudinal examination of urban areas in three African countries

**DOI:** 10.1186/s12884-015-0729-3

**Published:** 2015-11-11

**Authors:** Ilene S. Speizer, Peter Lance

**Affiliations:** Department of Maternal and Child Health, Gillings School of Global Public Health, The University of North Carolina at Chapel Hill, Chapel Hill, NC USA; Carolina Population Center, University of North Carolina at Chapel Hill, Chapel Hill, USA

**Keywords:** Fertility, Family planning, Africa, Urban, Unintended pregnancy, Intentions

## Abstract

**Background:**

Many women have inconsistent fertility desires and contraceptive use behaviors. This increases their risk of unintended pregnancies. Inconsistencies may reflect barriers to family planning (FP) use but may also reflect ambivalence toward future childbearing. Using urban data from Kenya, Nigeria, and Senegal, this study examines the role of fertility desires and FP use behaviors on pregnancy experience over a 2-year follow-up period.

**Methods:**

Data come from baseline and 2-year follow-up among urban women interviewed in Kenya, Nigeria, and Senegal. At baseline (2010/2011), women were asked about their future fertility desires (want child soon, want to delay >2 years, does not want) and current FP use. At midterm (2012/2013), women were asked if they were currently pregnant or had a birth in the 2-year period. We examine the association between baseline fertility desires and FP use with pregnancy experience and desirability of an experienced pregnancy.

**Results:**

In the 2-year follow-up period, 27–39 % of women in union experienced a pregnancy or birth. In Kenya and Nigeria, 30–35 % of women using a modern FP method experienced a pregnancy/birth; the percentage with a pregnancy/birth was slightly higher among women not using at baseline (41 % in both countries). In Senegal, the distinction between pregnancy experience between users and non-users was greater (16 % vs. 31 %, respectively). In all countries, pregnancy was less common among users of long-acting and permanent methods; only a small percentage of women use these methods. Women not wanting any(more) children were the least likely to experience a pregnancy in the 2-year follow-up period. No differences were observed between those who wanted to delay and those who wanted soon. Multivariate findings demonstrate distinctions in pregnancy experience by fertility desires among modern FP users. Non-users have similar pregnancy experience by fertility desires.

**Conclusions:**

Fertility desires are not stable; providers need to consider the fluidity of fertility desires in counseling clients. Programs focusing on new FP users may miss women who are the most motivated to avoid a pregnancy and need to switch to a more effective method; this will result in less unintended pregnancies overall.

## Background

Reducing unintended pregnancies is a central reproductive health goal, especially since unintended pregnancies are associated with poor birth and child health outcomes, unsafe abortion and maternal mortality [1–3]. Sub-Saharan Africa has some of the highest maternal mortality and unsafe abortion rates [4, 5] and some of the lowest contraceptive use rates [5]. Contraceptive use reduces the risk of unintended pregnancy while at the same time conveying other benefits including reduced recourse to (often) unsafe abortion, reduced maternal mortality, reduced neonatal, infant, and child mortality, and improved education and employment opportunities of women and men who delay initiation of childbearing [1, 6].

Unintended pregnancies are those pregnancies that are mistimed, that is they came sooner than desired or are unwanted (i.e. were not wanted at all). Prevention of unintended pregnancies requires that women have access to and control over the use of safe and effective family planning (FP) methods [7]. Recent global estimates suggest that about a third of all pregnancies in sub-Saharan Africa are considered unintended (mistimed or unwanted) with the percentage varying by region [8]. A little more than half (55 %) of all pregnancies in Southern Africa are unintended, while the figures are about 44 % in Eastern Africa and roughly a quarter in Northern and Western Africa [8]. The high prevalence of unintended pregnancies perhaps reflects the quarter of sexually active women in sub-Saharan Africa who report a desire to delay or limit childbearing but are not using an effective method of FP to meet these needs [9].

To examine the extent of unintended pregnancy at the aggregate level, demographic surveys often ask women about the intentionality of their last pregnancy or live birth. Typically, respondent women are asked, “At the time you became pregnant, did you want to become pregnant then, did you want to wait until later, or did you not want to have any(more) children at all?” A more nuanced way of examining pregnancy intentions and unintended pregnancy risk is to examine women’s motivations to avoid a pregnancy and how this corresponds to modern contraceptive use and subsequent pregnancy experience. This type of analysis has been performed in a small number of studies demonstrating that among women who report that they do not want any (more) children, a sizeable proportion (between 16–29 %) become pregnant in a 2 to 5 year follow-up period [10–17]. For example, in a study from Upper Egypt, Casterline and colleagues [17] demonstrate that among women who have an unmet need for limiting (that is they do not want any more children but are not using a FP method), 40 % became pregnant in the 2-year survey interval. Notably, only 12.5 % of women with a met need to limit (that is, women who do not want any more children and are using a FP method to avoid future childbearing) became pregnant in the follow-up period. This suggests that FP use is effective at reducing unintended pregnancy risk, but not completely, since women who were using a method to avoid a pregnancy contributed about 17 % of the unwanted pregnancies in the follow-up period [17].

The Upper Egypt study examines unmet need and unintended fertility. Unmet need is a standard measure used to determine gaps in FP services and is a key indicator of the FP2020 initiative [18]. At the aggregate level, unmet need is generally a rather stable indicator [17, 19], while at the individual level there is much change in unmet need classification even over short periods of time [16, 17, 19]. The individual-level instability of unmet need relates to two factors: women’s fertility desires are fluid and not stable, and women may start and stop contraceptive use.

There are a number of reasons that women may change their fertility desires and family planning use behaviors. Some of these relate to changes in circumstances through marriage, partner absence, and the death of a child. Furthermore, women may start and stop family planning use based on challenges with access (e.g., stock-outs; getting to the facility), costs of methods, quality of care (e.g., not receiving full information on resupply/re-injection), or fear of or experience with side effects [20, 21]. In addition, fertility intentions and family planning use behaviors are dependent on both the woman and her partner’s desires and behaviors and may be influenced by the cultural context within which they live. Changing intentions may thus reflect a variety of individual, interpersonal, and environmental influences on attitudes and behaviors [7, 21, 22]. Furthermore, it is possible that women (and partners) are ambivalent about future childbearing. This ambivalence can lead to non-use and thus exposure to a pregnancy (intended or unintended) [23].

The current FP2020 Initiative focus on reducing unmet need and identifying 120 million new FP users by 2020 [24] may miss the mark by ignoring current users who are motivated to avoid a pregnancy and need a more effective method [17, 25]. These women may be the most likely to resort to abortion if they experience contraceptive failure and an unintended pregnancy [10]. Moreover, even women who appear to have an unmet need may not be the most motivated to adopt a method if they are ambivalent about future childbearing [16, 26].

In this paper, we use recently collected longitudinal data from a 2-year follow-up period for women surveyed in urban Kenya, Nigeria, and Senegal to demonstrate the extent to which contraceptive use and fertility desires are associated with subsequent pregnancy experience. The paper is in part motivated by a recent increase in attention to urban areas since much of the rapid growth in urban areas comes from high fertility, suggesting a continued need to address FP needs in urban environments, particularly among the poor and uneducated who tend to have the highest fertility [27]. This paper focuses on major urban areas in three sub-Saharan African countries to provide insights into fertility experience, unmet need, and gaps in FP services in the study urban areas.

### Study contexts

The three sub-Saharan African countries used in this study have varying fertility and family planning contexts and thus provide rich settings to explore associations between fertility desires and family planning use. Nigeria is the largest of the three countries and is the most urban as well with roughly 50 % of Nigeria’s population living in urban areas. Senegal has about two-fifths of its population living in urban areas while Kenya is only one quarter urban [28]. Information from the Demographic and Health Surveys is available from the three countries on key indicators that relate to this study. Comparing urban data from the three countries, urban Nigeria (2013) had the highest fertility at 4.7 children per woman, the lowest modern contraceptive use (14.9 %) and the lowest percentage of women that want no more children [29]. The urban context for Senegal (2014) has a high total fertility rate (4.0 children per woman), about one-fifth of women that want no more children (19.5 %) and a little more than a quarter of women are using modern contraception (28.8 %) [30]. The Kenya urban context is different from the other two countries with lower fertility (3.1 children per woman) and a high percentage of women that want no more children (47 %) and a high percentage currently using modern contraception (57 %) [31].

## Methods

This study uses baseline and 2-year follow-up data from a longitudinal sample of women in three or four cities in three countries: Kenya (Nairobi, Mombasa and Kisumu); Nigeria (Abuja, Ibadan, Ilorin and Kaduna); and Senegal (Guédiawaye, Pikine and Mbao)^1^. The data were collected as part of the Measurement, Learning & Evaluation (MLE) Project’s evaluation of the Urban Reproductive Health Initiative, a Bill & Melinda Gates Foundation-funded program in four countries (the fourth site is the state of Uttar Pradesh, India) with the goal of increasing modern contraceptive use in urban areas. Details of the programs and the data collection methods can be found elsewhere [32].

At baseline (2010/2011), in each city a two-stage sampling design was used to select a representative sample of women at baseline. In the first stage, a random sample of primary sampling units (PSUs) was selected based on a recent census sampling frame. In the second stage, all households in selected PSUs were listed and mapped and a random sample of those households was selected for interview. All women (ages 15–49) in selected households were eligible for interview following the informed consent process. At baseline, women were asked about their knowledge, ever use, and current use of FP as well as their future fertility desires. In each country, probability weights were used to adjust the multi-city sample to be representative across the cities.

Two-years after baseline data collection (midterm – 2012/2013), all women in the three study cities in Kenya and Senegal and a random sub-sample of women in the four study cities in Nigeria were tracked and asked to participate in a follow-up interview^2^. Midterm interviews obtained information about births in the follow-up period as well as about contraceptive use since the time of the baseline interview. At midterm, women were asked for their informed consent to participate. The midterm probability weights were designed to render the midterm sample longitudinally representative and adjusted for attrition.

In all three countries, all women ages 15–49 were asked to provide consent to participate; in Senegal, this consent was a written consent process whereas in Kenya and Nigeria this was a verbal consent process. As done with other large-scale demographic surveys in these countries, no parental consent was required for women under the age of 16. Ethical approval for the study protocol and informed consent process (waiver of parental consent of minors and waiver of signed consent in Nigeria and Kenya) was obtained from the Institutional Review Board at the University of North Carolina at Chapel Hill and the Kenya Medical Research Institute Ethical Review Committee (for Kenya), the National Health Research Ethics Committee of Nigeria (for Nigeria), and the Comité National d’Ethique pour la Recherche en Santé in Senegal (for Senegal).

To examine whether baseline fertility desires and contraceptive use are associated with subsequent pregnancy experience, this analysis focuses on those women who are fecund and non-sterilized at baseline. The small number of women who self-reported on the future fertility desires question that they could not have any more children (infecund) and those who were sterilized at baseline were dropped. All remaining women are included in the analysis that determines if they had a birth or pregnancy in the 2-year follow-up period since the baseline interview. Analyses are presented for all women and for women in-union; the focus on women in union is particularly relevant in the Nigeria and Senegal settings where most reported sexual activity takes place in union.

The key outcome variable of interest to this analysis is whether the woman had a pregnancy or birth between the baseline and midterm surveys. This variable is coded one if the woman is currently pregnant at midterm or if she had a birth since baseline; all others with no pregnancy/birth are coded zero. All women who were currently pregnant at endline were asked if the current pregnancy was wanted then, wanted later, or not wanted at all. Likewise, women who had a birth in the 2 years since baseline were asked whether the most recent birth was wanted then, wanted later, or not wanted at all. If a woman was currently pregnant and had a prior birth in the 2-year follow-up period, for this analysis we used the information on the intentions of the current pregnancy. Using these two questions we create a three category variable of intentionality of the last pregnancy/birth: wanted then, wanted later, not wanted. The wanted later and not wanted are unintended pregnancies; wanted later are mistimed pregnancies and not wanted are unwanted pregnancies.

For this analysis, we use women’s baseline contraceptive use and fertility desires as the key independent variables of interest. At baseline, all women were asked if they or their partner were currently doing something to avoid getting pregnant. Women who reported that they were using a method were asked what method they were using. Women using multiple methods were classified as users of the most effective method they were using. At baseline, traditional methods included withdrawal, rhythm, and standard days method^3^ and modern methods included the intrauterine device (IUD), implants, injectables, oral contraceptive pill, male and female condoms, and lactational amenorrhea (LAM). For descriptive analyses, we group women into four categories based on their baseline method use: non-user, long acting method users (IUD and implants), spacing method users (injectable, oral contraceptive pill, male and female condoms, and LAM), and traditional method users. For multivariate analyses, we examine modern method users versus all other women.

The second key independent variable is fertility desires at baseline. All women were asked, “In the future do you want any(more) children?” Women who reported yes were asked how long they would like to wait to have the next pregnancy. Among women who were pregnant at baseline, they were asked about their desire for a subsequent pregnancy. Women were coded as a) wants another pregnancy now (i.e., within 2 years), b) wants another pregnancy after 2 years, c) wants a pregnancy after marriage, d) wants but does not know when, and e) does not want (any) more. The small number of women who gave open responses to these questions was recoded into appropriate categories if feasible. For example, women who reported that they wanted to wait until they finished school or earned enough money were coded into the category for wants to delay more than 2 years. Those women who reported that they wanted a child but for timing they said “up to God” or some other answer without clear timing were coded as wants but do not know when. Finally, those who reported “when remarried” or some other answer related to marriage were recoded as wants a pregnancy after marriage. For the multivariate analyses, the women who reported that they wanted more children but did not know when were dropped from the analysis. For the multivariate analyses of women ever in union, the small number of women who inconsistently reported “wants a pregnancy after marriage” was dropped from the analyses.

All analyses control for key demographic factors that are associated with fertility and FP outcomes. The control variables included are: city, parity, employment in the last year, age group, religion, education level, wealth, and marital status (for all women model). Wealth was created as wealth quintiles using principal components analysis as done in large, demographic surveys [33]. Distributions of these variables are presented in Table 1 for the interviewed and analysis samples.

**Table 1:** Percentage of eligible women interviewed, characteristics of women not inteviewed and interviewed and characteristics of analysis sample by country

	Kenya			Nigeria			Senegal		
	Percent interviewed of eligible sample^a^	Distribution among women interviewed^a^	Analysis sample^b^	Percent interviewed of eligible sample^a^	Distribution among women interviewed^a^	Analysis sample^b^	Percent interviewed of eligible sample^a^	Distribution among women interviewed^a^	Analysis sample^b^
City									
Nairobi/Abuja/Guédiawaye	48.8	68.9	74.2	57.9	15.5	17.7	78.7	25.0	24.7
Mombasa/Ibadan/Pikine	64.7	25.2	20.8	64.6	26.2	27.8	79.5	26.3	25.2
Kisumu/Ilorin/Mbao	57.9	5.9	5.0	64.7	23.0	21.9	74.2	48.7	50.1
na/Kaduna/na	na	na	na	65.1	35.3	32.6	na	na	na
Age group									
15–19	41.8	9.4	14.1	62.8	16.2	17.6	75.2	20.8	24.2
20–24	45.7	25.1	30.6	53.0	15.2	18.9	72.5	20.5	20.1
25–29	51.6	23.6	22.2	59.5	18.7	19.1	74.2	16.5	16.7
30–34	57.6	16.0	15.7	64.4	16.5	17.6	79.3	14.1	13.6
35+	65.7	25.8	17.5	73.2	33.5	26.8	81.4	28.1	25.4
Marital status									
Never married	39.5	25.7	33.2	56.1	30.1	31.9	77.4	38.4	42.3
Ever married	59.5	74.2	66.8	67.5	69.6	68.1	76.2	61.5	57.7
Missing	21.4	0.1	0.0	66.0	0.3	0.0	70.7	0.1	0.0
Parity (mean)	na	1.9	1.6	na	2.5	2.3	na	2.1	1.9
Education									
None/quaranic	57.3	3.8	3.2	66.1	10.1	9.5	70.3	32.7	31.9
Primary	53.0	40.1	40.4	64.7	13.8	13.6	78.8	37.1	37.1
Secondary	53.3	39.8	38.5	64.0	48.5	50.4	82.7	27.5	27.8
Higher	49.0	16.3	17.8	61.6	26.7	26.5	73.2	2.7	3.2
Missing	0.0	0.0	0.0	66.7	0.9	0.0	0.0	0.0	0.0
Religion									
Catholic	51.7	22.0	22.3	64.6	5.0	5.6	81.0	7.2	11.5
Protestant	51.8	63.9	65.3	61.0	42.0	43.8	81.7	1.1	1.8
Muslim	58.0	12.0	10.6	65.6	52.2	50.0	76.2	91.6	86.3
None/other/miss	55.3	2.1	1.9	80.9	0.7	0.6	93.0	0.1	0.4
Wealth									
Poorest	51.9	16.8	16.8	53.1	14.5	16.4	64.4	13.7	14.3
Poor	54.3	19.7	19.3	63.7	17.9	18.0	81.6	22.3	21.3
Middle	56.9	21.3	19.4	66.3	20.0	19.5	79.7	27.1	26.7
Rich	54.6	21.7	20.3	67.9	22.4	21.2	79.1	20.6	20.5
Richest	46.2	20.6	24.2	65.4	25.2	24.9	74.5	16.3	17.2
Worked in last 12 months	53.2	62.8	61.2	66.5	54.6	54.0	77.5	15.8	15.6
Total/Weighted counts	52.5	3007	3108	63.6	5008	4001	76.6	2044	2721

Nigeria - midterm eligible sample was 65 % of baseline sample in four cities; Kenya and Senegal - full sample eligible in three cities

^a^Uses baseline weights

^b^Excludes women that were sterilized or infecund at baseline; uses midterm weights

Univariate, bivariate, and multivariate analyses are presented in this paper. The presentation of the sample attrition uses baseline weights, however, all other analyses use midterm weights of the analysis sample. All analyses adjust for the clustering of the data using svy commands in Stata statistical software. Interactions are included in the multivariate models. Results from interactions are presented using marginal estimates to facilitate interpretation of the results. The interactions lend themselves to the calculation of conditional odds ratios. For instance, interacting contraceptive use with intentions (as we do below) allows us to calculate the odds ratios for intentions conditional on use and non-use of contraception.

To clarify the estimates we present, consider a simple example involving two binary independent variables, *x*_1_ and *x*_2_, and an outcome *D*. We perform logistic regression of $D$ on *x*_1_ and *x*_2_ and the interaction of *x*_1_ and *x*_2_. The conventional logistic probability that D = 1 is

$$ Pr\left(D=1\left|{x}_1,{x}_2\right.\right)=\frac{exp\left({\beta}_0+{\beta}_1\cdot {x}_1+{\beta}_2\cdot {x}_2+{\beta}_3\cdot {x}_1\cdot {x}_2\right)}{1+ exp\left({\beta}_0+{\beta}_1\cdot {x}_1+{\beta}_2\cdot {x}_2+{\beta}_3\cdot {x}_1\cdot {x}_2\right)} $$

The conventional odds ratios for the terms *x*_1_, *x*_2_ and *x*_1_ ⋅ *x*_2_ are, respectively,

$$ \begin{array}{ccccc}\hfill \hfill & \hfill \hfill & \hfill \hfill & \hfill \hfill & \hfill exp\left({\beta}_1\right)\hfill \\ {}\hfill \hfill & \hfill \hfill & \hfill \hfill & \hfill \hfill & \hfill exp\left({\beta}_2\right)\hfill \\ {}\hfill \mathrm{and}\hfill & \hfill \hfill & \hfill \hfill & \hfill \hfill & \hfill \hfill \\ {}\hfill \hfill & \hfill \hfill & \hfill \hfill & \hfill \hfill & \hfill exp\left({\beta}_3\right)\hfill \end{array} $$

However, the presence of the interaction terms suggests another set of interesting odds ratios:

$$ exp\left({\beta}_1+{\beta}_3\right) $$

is the odds ratio associated with *x*_1_ when *x*_2_ = 1 while

$$ exp\left({\beta}_1\right) $$

is the odds ratio associated with *x*_1_ when *x*_2_ = 0. The later set of odds ratios offer us the opportunity to see how the overall odds ratio for one variable depends on the value of another variable. These are the results presented and interpreted for the final models.

## Results

Table 1 presents the descriptive characteristics of the midterm sample including the percentage of eligible women interviewed in each country, the characteristics of women interviewed, and the characteristics of the analysis sample (women fecund and not sterilized at baseline). The highest midterm response rates across the three countries were in Senegal, where 77 % of the baseline eligible sample was interviewed. In Nigeria and Kenya the figures are 64 and 53 %, respectively. Not surprisingly, the response rate differs by some demographic characteristics. In particular, in Kenya, a greater percentage of women from Mombasa were surveyed followed by Kisumu; Nairobi had the lowest response rates. In Nigeria, the lowest response rates were also found in the capital, Abuja, and the other three cities were similar at about 65 %. Response rates were generally higher for older women across the three countries. In Kenya and Nigeria, response rates were higher among women who at baseline were ever married as compared to women never married.^4^

The second column for each country provides the distribution of the sample that was interviewed at midterm for the full sample. Finally, the last column in Table 1 for each country presents the distribution of the demographic characteristics for the analysis sample of women who were fecund and not sterilized at baseline; results in this column use midterm weights to adjust for non-response. These are the women who are the focus of the analyses that follow. As expected, this group is slightly younger and has lower parity than the distribution among the full sample surveyed at midterm.

Table 2 provides the weighted distribution of the key independent variables in the analysis sample of all women and in the smaller sample of women ever in union at baseline. As found with the DHS data, the data in Table 2 demonstrate low urban modern method use, particularly in Nigeria and Senegal, and a large desire to delay or limit childbearing. The values shown here are slightly different than the DHS urban data reported earlier; this is a consequence of the MLE baseline data taking place in 2010 and the MLE data focusing on major urban areas and not a random sample of urban areas. As expected, women in the ever in union sample report greater current use of a modern family planning (FP) method at baseline. This is expected because this sample is more likely to be sexually active. Use is highest in Kenya with 41 % of women using a modern method; among women ever in union in Kenya, modern method use is higher at 51 %. About a quarter of women (all women and women ever in union) are using a modern method in Nigeria at baseline. In Senegal, only 15 % of all women and a quarter of women in union are using a modern method at baseline. Across all countries, the method mix is similar with the majority of use being spacing methods (injection, pill, and condom). Nigeria has the highest use of traditional methods while Kenya has the highest use of long-acting methods (IUD and implant).

**Table 2:** Distribution of key independent variables at baseline among full analysis sample and among analysis sample that is ever in union at baseline

	Kenya analysis sample		Nigeria analysis sample		Senegal analysis sample	
	Full	Ever in union	Full	Ever in union	Full	Ever in union
% using modern method at baseline	41.4	51.4	23.6	27.5	14.9	24.8
Method mix at baseline						
Non-user	54.7	44.1	68.3	61.9	83.5	72.5
LAPM (not sterilization)	4.8	6.3	3.0	4.3	1.5	2.4
Spacing method	36.7	45.1	20.7	23.2	13.4	22.4
Traditional method	3.9	4.5	8.1	10.6	1.5	2.6
future intention at baseline						
Want another now	13.6	15.4	18.2	24.4	17.8	28.1
Want to delay	39.6	34.9	25.1	29.5	26.7	41.4
Want after marriage	5.4	0.2	16.4	0.4	38.0	1.0
Wants don’t know when	8.0	8.0	17.7	13.6	4.6	7.1
No more	33.3	41.5	22.6	32.1	12.9	22.4

Also presented in Table 2 are the baseline future fertility intentions among the full and ever in union samples. In the full sample, more than a third of women in Senegal report that they want a child after marriage; in the Senegalese context there are low levels of women reporting premarital sex. In Nigeria, 16 % of women report that they want a child after marriage while only 5 % of women in Kenya provide this response. Among all women and women ever in union in all three countries, a common response is that the woman wants children but also wants to delay a pregnancy 2 or more years. Among women in Kenya, a third of all women and two-fifths of women in union do not want any(more) children. In Nigeria, between a quarter and a third give this response whereas in Senegal, only 13 % of all women and 22 % of women ever in union report that they do not want any(more). Senegal and Nigeria also have about a quarter of women in union who want a child now (soon). These data indicate unmet needs for family planning given that the sum of the percentages who want to delay or limit childbearing is at least 24 % larger than the percentage of women that are currently using a modern method of FP in each of the samples.

Table 3 presents the percentage of women who experienced a pregnancy or birth between baseline and midterm by baseline modern FP use and fertility intentions. Across the three countries, between 18–30 % of all women surveyed and 27–39 % of ever in-union women surveyed experienced a pregnancy in the 2-year follow-up period. In all three countries, about 15–30 % of women using modern FP at baseline became pregnant between the surveys. Interestingly, among all women in Kenya and Nigeria, the percentage that experienced a pregnancy among those not using a modern FP method at baseline (29–30 %) is similar to the percentage of women using that became pregnant. In Senegal, a slightly higher percentage of those not using at baseline became pregnant (18 % vs. 15 %). The values for pregnancy experience by baseline contraceptive use among women ever in union are slightly higher for non-users than users in Kenya and Nigeria (41 % among non-users in both countries vs. 30 and 35 % for users, respectively). In Senegal, among women ever in union, only 16 % of users of a modern method became pregnant in the 2-year follow-up period compared to 31 % of non-users in union.

**Table 3:** Percentage of women who experienced a pregnancy between baseline and midterm by baseline method use, baseline intentions, and intentionality of the pregnancy/birth at midterm

	Kenya		Nigeria		Senegal	
Baseline union status	All women	In union	All women	In union	All women	In union
	% pregnant	% pregnant	% pregnant	% pregnant	% pregnant	% pregnant
Baseline modern method use						
No	29.4	40.5	29.6	41.1	18.3	31.2
Yes	28.2	29.8	29.6	35.0	15.5	16.2
Baseline method use						
Non-user	29.2	41.0	29.6	42.6	29.2	41.0
Long acting method	17.7	14.9	20.0	20.1	17.7	14.9
Spacing method	29.5	31.9	30.9	37.8	29.5	31.9
Traditional method	32.7	35.7	29.1	32.0	32.7	35.7
Baseline intentions						
Want another now	47.2	53.1	45.0	47.2	24.1	24.9
Want to delay	35.1	48.3	51.3	60.8	33.8	38.0
Want after marriage	15.8	na	5.9	na	4.8	na
Wants don’t know when	27.4	35.1	23.2	37.6	20.6	22.6
No more	16.6	17.0	15.1	14.6	11.8	11.9
% pregnant	28.9	35.0	29.6	39.4	17.9	27.4
Total n	3108	2072	4001	2713	2721	1554

The main difference in the probability of a pregnancy between surveys is seen by type of method. Those women who are using long acting methods are about half as likely to have experienced a pregnancy or birth between baseline and midterm as non-users and women using spacing or traditional methods. Notably, only a small percentage of women in all countries use these most effective methods (see Table 2).

Also presented in Table 3 is the percentage of women who became pregnant between baseline and midterm by baseline fertility intentions. Among women ever in union, between 25 % (Senegal) and 53 % (Kenya) of women who wanted a child soon/now became pregnant. In Nigeria and Senegal, the highest percentage with a pregnancy in the 2-year follow-up period was among women ever in union who wanted to delay 2 or more years (61 and 38 % of women who want to delay become pregnant in Nigeria and Senegal, respectively). Among women ever in union who said they wanted a child but didn’t know when, between 23 % (Senegal) and 38 % (Nigeria) became pregnant. Finally, among women in union who wanted no more children, 12 % of women from urban Senegal became pregnant; the corresponding values for Nigeria and Kenya were 15 and 17 %, respectively. This supports the notion that fertility intentions are meaningful but not deterministic.

Table 4 presents a summary of the multivariable logistic regression results for pregnancy experience between baseline and midterm for the women ever in union; results were similar for the all women models (contact first author for copies of those results). All models control for employment in the last year, parity, age group, religion, education level, city, and wealth (not shown). Model 1 shows the results with no interactions while Model 2 includes an interaction between baseline modern family planning use and baseline fertility desires. At the top of Model 2, we present the regression results while at the bottom the interacted odds ratios are presented for each intention and FP use group for ease of interpretation.

**Table 4:** Multivariate regression findings of factors associated with pregnancy experience over the 2-year follow-up period, Model 1 without interaction, Model 2 with interaction

	Kenya women ever in union		Nigeria women ever in union		Senegal women ever in union	
	Model 1	Model 2 (interactions)	Model 1	Model 2 (interactions)	Model 1	Model 2 (interactions)
Baseline fertility intentions						
Wants now/within 2 years	1	1	1	1	1	1
Wants in +2 years	.65 (.40,1.05)*	1.50 (.80,2.78)	1.12 (.87,1.43)	1.32 (.97,1.78)*	1.52 (1.07,2.15)**	1.82 (1.17,2.81)***
Does not want	.30 (.18,.50)***	.70 (.38,1.26)	.44 (.31,.63)***	.58 (.39,.87)***	1.05 (.50,2.22)	1.46 (.61,3.53)
Baseline modern method use						
Non-user	1	1	1	1	1	1
User	.48 (.33,.69)***	2.94 (1.10,7.87)**	.89 (.68,1.16)	1.85 (1.05,3.26)**	.31 (.19,.51)***	.98 (.33,2.96)
Interaction terms						
Use *Wants in +2 years		.12 (.04,.37)***		.43 (.22,.82)**		.28 (.07,1.12)*
Use *Does Not Want		.10 (.03,.31)***		.32 (.13,.79)**		.12 (.01,1.05)*
Interacted odds ratios						
Baseline fertility intentions | modern use = 1						
Wants now/within 2 years		1		1		1
Wants in +2 years		.17 (.07,.43)***		.57 (.34,.95)**		.51 (.17,1.58)
Does Not Want		.07 (.03,.18)***		.19 (.08,.41)***		.17 (.02,1.23)*
Baseline fertility intentions | modern use = 0						
Wants now/within 2 years		1		1		1
Wants in +2 years		1.50 (.80,2.78)		1.32 (.97,1.78)*		1.82 (1.17,2.81)***
Does not want		.70 (.38,1.26)		.58 (.39,.87)***		1.46 (.61,3.53)

*p < 0.10; **p < 0.05; ***p < 0.01

Focusing first on Kenya, Model 1 shows that modern FP users are less likely to get pregnant (OR: 0.48; 95 % CI: (0.33, 0.69)). In addition, among women who do not want any(more) children, there is a lower odds of a pregnancy in the follow-up period (OR: 0.30; 95 % CI: (0.18, 0.50)). In Model 2, both interactions are significant indicating that FP use and intentions jointly matter. In particular, among FP users at baseline, those who do not want any(more) children are the least likely to get pregnant (OR: 0.07; 95 % CI: (0.03, 0.18)), followed by users who want to delay 2 or more years (OR: 0.17; 95 % CI: (0.07, 0.43)); each of these user groups has a lower odds of a pregnancy than users who want to get pregnant soon. Further, among non-users, there is no difference in pregnancy experience in the 2-year follow-up by baseline intentions.

For Nigeria, Model 2 shows that among users, those who do not want any(more) children have significantly lower odds of a pregnancy than users who want soon (OR: 0.19; 95 % CI: (0.08, 0.41)). Likewise, users who want to delay childbearing are also significantly less likely to experience a pregnancy in the follow-up period (OR: 0.57; 95 % CI: (0.34, 0.95)). Unlike Kenya, among non-users in Nigeria, we find that those women who report that they do not want any(more) children are the least likely to experience a pregnancy in the follow-up period (OR: 0.58; 95 % CI: (0.39, 0.87)). Conversely, non-users who report a desire to delay childbearing 2 or more years were the most likely to experience a pregnancy in the follow-up period (OR: 1.32; 95 % CI (0.97, 1.78)). This may reflect higher fecundability among women who want to delay as compared to women who want now and may have been trying to get pregnant for a long time.

Finally, for women ever in union in urban Senegal, Model 2 shows that among modern method users, the main distinction in pregnancy experience is among those who report that they do not want any(more) children; these women were less likely (*p* < 0.10) to become pregnant in the follow-up period (OR: 0.17; 95 % CI: (0.02, 1.23)). Similar to what was found in Nigeria, among non-users in Senegal, those who report at baseline that they want a pregnancy after 2 years were significantly more likely to experience a pregnancy in the follow-up period than those who were non-users and wanted a pregnancy immediately (OR: 1.82; 95 % CI: (1.17,2.81)).

Table 5 presents the cross-tabulations of baseline fertility desires and midterm reported intentionality of the experienced pregnancy among women ever in union who became pregnant between baseline and midterm. Overall in Kenya, 58 % of pregnancies were reported as “wanted then” while nearly 40 % were reported as “unintended” (29 % mistimed and 11 % unwanted). In Kenya, among women who at baseline wanted to become “pregnant soon/now”, 86 % reported that the pregnancy was wanted then while only 14 % reported that the pregnancy came too soon or was unwanted. Among women who at baseline wanted to delay and women who wanted but didn’t know when, about 50 % reported that the pregnancy was wanted then and another 36–41 % reported that the pregnancy came too early (wanted to wait 2+ years). Among these women only 7–8 % reported that the pregnancy was not wanted. Among women who wanted no more children at baseline, more than two-fifths (46 %) reported that the pregnancy was wanted then. Notably, more than half of pregnancies among women who did not want any(more) at baseline were reported as unintended (23 % mistimed and 29 % unwanted).

**Table 5:** Reported intentionality of pregnancies at midterm based on baseline fertility desires among women ever in union who got pregnant between baseline and midterm

	Midterm reported intentionality of pregnancy					
Kenya	Wanted then	Wanted within 2 years	Wanted to wait >2 years	Did not want	Weighted number	Total
Baseline intentions						
Want another now	86.26	4.27	5.42	4.05	170	100 %
Want to delay	49.84	1.84	41.33	6.99	350	100 %
Wants don’t know when	53.11	2.18	36.37	8.34	59	100 %
No more	46.37	2.08	22.57	28.98	146	100 %
Total	57.9	2.5	28.7	10.8	725	100 %
	Midterm reported intentionality of pregnancy					
Nigeria	Wanted then	Wanted within 2 years	Wanted to wait >2 years	Did not want	Weighted number	Total
Baseline intentions						
Want another now	84.93	3.13	6.81	5.14	314	100 %
Want to delay	80.05	2.56	14.01	3.37	488	100 %
Wants don’t know when	83.79	1.59	6.71	7.91	139	100 %
No more	76.22	1.49	5.80	16.49	128	100 %
Total	81.5	2.5	10.0	6.1	1069	100 %
	Midterm reported intentionality of pregnancy					
Senegal	Wanted then	Wanted within 2 years	Wanted to wait >2 years	Did not want	Weighted number	Total
Baseline intentions						
Want another now	81.43	4.99	13.58	0.00	107	100 %
Want to delay	56.32	8.03	35.65	0.00	240	100 %
Wants don’t know when	50.93	6.24	42.82	0.00	25	100 %
No more	32.09	0.00	49.13	18.79	41	100 %
Total	60.1	6.3	31.7	1.9	413	100 %

The results for Senegal are similar to those of Kenya with the exception that only a small percentage of women who became pregnant at midterm reported the pregnancy as unwanted; this reflects a more pronatalist culture in Senegal. The results for Nigeria suggest that there is high ex-post rationalization and pro-natalist beliefs in the urban contexts under study. Among women who became pregnant, the overwhelming majority reported the pregnancy as wanted then. This was true among women who at baseline wanted immediately as well as among those who wanted to delay and those who wanted but didn’t know when. In addition, more than three quarters of women who indicated at baseline that they did not want any(more) children reported the experienced pregnancy as wanted now. Only 22 % of women who did not want any(more) children at baseline reported the pregnancy as unintended (mistimed – 6 %, or unwanted – 16 %).

## Discussion

The overall goal of family planning programs is to help women who want to delay or avoid childbearing to meet these stated fertility desires. This study demonstrates gaps between intentions to have a child and modern family planning use in urban areas of each of the three African countries considered. In particular, comparing the percentage of women who want to delay or limit childbearing with the percentage using modern methods in each country we see that a quarter to a third of women are likely in need of a method to meet their baseline fertility desires. Moreover, there is high fertility in the study cities given that between a quarter and a third of women in union experienced a pregnancy in the 2-year follow-up period. Notably, while experience of a pregnancy is lower among women in union who were using a method, the difference in pregnancy experience between users and non-users is not large. Between 16 % (Senegal) and 35 % (Kenya) of women in union who were using a modern method experienced a pregnancy in the 2-year follow-up period. This likely reflects use of spacing methods by women who want to delay birth. The small number of women using long-acting methods (IUD and implant) were the least likely to get pregnant.

Our findings also demonstrate that fertility intentions matter. Overall, women who do not want any(more) children were the least likely to experience a pregnancy. Conversely, in Nigeria and Senegal, women who wanted to delay pregnancy were the most likely to experience a pregnancy. The difference in pregnancy experience between women who wanted immediately and women who wanted to delay is notable. This may reflect lower fecundability among women who want immediately if these women have been trying to get pregnant for a while. Multivariate findings demonstrate that those women using a modern method who wanted no more children were the least likely to experience a pregnancy followed by those who were users and wanted to delay. Among non-users, intention to avoid childbearing only distinguished pregnancy experience in Nigeria.

Examination of the reported intentionality of experienced pregnancies demonstrated ex-post rationalization of experienced pregnancies. Most women who reported a desire to delay or to avoid childbearing reported an experienced pregnancy as wanted then, suggesting that fertility desires may not be firmly held or that women are willing and able to accept an unintended pregnancy in these pro-natalist cultures. Notably, 16 % of women from Nigeria, 19 % from Senegal, and 29 % of women from Kenya who became pregnant and reported at baseline that they did not want any(more) children reported the pregnancy as unwanted. These are the women that programs should be seeking to reach with the most effective methods to avoid these unwanted pregnancies and to support women to attain their fertility desires. Finally, while Kenya has the highest percentage of women who are using a modern method, they also have the highest percentage of pregnancies that are reported to be mistimed or unwanted (unintended). In this context of high access to and use of family planning methods, women who choose not to use may be ambivalent about future childbearing.

Our finding that between 12 % (Senegal) and 17 % (Kenya) of women in union who do not want any(more) children experience a pregnancy in the 2-year follow-up period is similar to other studies that examined fertility experience over time. For example, longitudinal studies from Nigeria and Morocco showed that 16 and 29 %, respectively, of women who did not want any(more) children had a birth in the 2-year follow-up period [10, 13]. Two studies with 5-year follow-up periods, one in France and the other in Bangladesh, found a similar percentage (17–20 %) of women who did not want any(more) children that became pregnant in the follow-up period; this may reflect more effective family planning use over the 5 year period [11, 14]. In relation to this, a number of studies have demonstrated that fertility desires are fluid and not stable [16, 34]. Our results likely reflect the fluidity of fertility desires (as well as high overall fertility) in generally pro-natalist cultures. Moreover, prior research has demonstrated that fertility desires are multi-dimensional and there is a need to consider contextual, partner, and cultural influences on fertility and family planning behaviors [35, 36].

Furthermore, women’s use of family planning may also be fluid. In particular, given that injections are the most common method used in all three of the African countries studied, it is not surprising that many women who were using a method became pregnant. Many women incorrectly use injections, including starting and stopping the method because of side-effects, missing a follow-up injection or delaying a follow-up injection [37]. Users of injectable methods may be ambivalent about a subsequent pregnancy and ambivalent about use of the injection (or any method) [38]. In pro-natalist contexts with high discontinuation of family planning methods, many women who say they want to delay or avoid a pregnancy may never adopt a method or may not continuously use a method and thus will be in the unmet need category.

In the 2 year follow-up period, the Urban Reproductive Health Initiative programs were launched with the goal of increasing modern family planning use in urban areas in the three countries studied. These programs sought to increase demand for family planning using various media outlets (e.g., radio, television, and print media) to demonstrate the benefits of family planning and promote couple discussion about family planning. It is expected that these types of activities would have made women more effective users. However, it may have also led women to have more frequent discussions on family planning use with their partners which could lead to discontinuation of a method should the partner not support continued use. Further analyses are required to better understand the role of these intervention activities on pregnancy experience, fertility desires, and subsequent family planning use; these types of analyses will be performed using the 4-year endline follow-up data for the three countries where women have a longer period to experience the relevant transitions.

This study has a number of strengths that are worth noting. First, by using longitudinal data in each of these countries, we are able to examine the association between baseline intentions and baseline contraceptive use on women’s pregnancy experience. Second, we have a short follow-up period, reducing the possibility of recall bias and the likelihood of multiple changes in fertility intentions over a longer period of time. Third, by including women from three African countries with a focus on urban areas, we can show the similarities across the results in the three countries but also note distinctions. For example, Kenya has the highest modern FP use but also the highest percentage of pregnancies reported as unintended.

This study is not without limitations. First, it is possible that fertility experience is higher in this sample than in a fuller sample that would include the women who were not found at midterm. In particular, if the mobile and missed women had less pregnancy experience, this would suggest that the overall percentage pregnant would be lower, should they have been found. Second, the loss to follow-up is high, particularly in Kenya and Nigeria. We use study weights to adjust for this loss to follow-up, using baseline data to identify strategies to weight the data to be representative of the baseline sample. Finally, because the focus of this paper is on current pregnancies and live births, we did not include reported abortions since 2010. It is possible that some of the women who had unintended pregnancies (i.e., reported a desire to avoid or delay childbearing but became pregnant) had an induced abortion and this information would not be captured by this analysis which focused on current pregnancies or live births as reported by the women. Information on abortion experience was available in the midterm survey; however, we think there was under-reporting of abortions and thus do not see this as contributing to the outcome of interest. Future studies with a larger sample size may be able to better measure abortion experience and the contribution of abortion to unintended pregnancies and the fluidity of fertility desires.

## Conclusions

To conclude, this is the first study to examine the longitudinal association between fertility desires and family planning use with later pregnancy/birth experience in urban African settings. As mentioned earlier, urban areas have higher access to and use of family planning and thus could be considered to be low priority for family planning program efforts. Our results show that about a quarter of women in the cities of the three countries included has inconsistent fertility desires and family planning use behaviors (i.e., an unmet need for family planning). These women are at a high risk of experiencing a pregnancy in the near future. That said, many of these pregnancies will be considered as intended, even among women who reported that they do not want any(more) children. Thus, we demonstrate that fertility desires are not static and programs should consider strategies to assess women’s (and couple’s) short- and long-term fertility desires at the time of counseling on family planning. Programs to train providers to do motivational counseling (i.e., tailoring counseling strategies to women’s specific fertility desires and partnership dynamics – [39]) may fill this need; these types of targeted programs will ensure that clients’ are receiving appropriate methods to meet their needs including long-acting methods for those women who report that they don’t want any(more) children. Furthermore, providers should be trained on effective counseling on side effects and effective use of spacing methods to ensure that users of these methods are able to meet their current and future fertility desires.

The FP2020 strategy that seeks to attain 120 million new users by 2020 may succeed in getting women (and couples) to adopt a method, but these women (and couples) may not be motivated users and this may lead to high discontinuation and negative attitudes and myths about family planning. Governments, funders, and program planners seeking to meet FP2020 goals should identify strategies to get motivated new users rather than any new user. This may include supporting women to switch from less effective methods to more effective methods that better meet their fertility and FP needs. While getting women to switch methods will not lead to more new users, it will, however, result in less unintended pregnancies which is ultimately the goal of any family planning and reproductive health program.

## Footnotes

1 In Nigeria and Senegal, baseline data were collected in six cities and in Kenya five cities; the smaller set of cities listed here had midterm data collected to inform program improvements at midterm and thus are the focus of this analysis.

2 In Nigeria, all women were actually tracked in the study cities while only those living in a randomly selected 65 percent of the baseline PSUs were eligible for interview.

3 Standard days method (SDM) was grouped with traditional methods at baseline and thus it is not possible to pull it out as a separate method in these analyses; in later surveys, SDM was a separate category.

4 Note that field teams felt that there were many women at baseline who were house girls (i.e., unmarried) who had moved and were not possible to track at midterm.
